# Case report of QT interval prolongation induced by anamorelin in an obese patient with non-small cell lung cancer

**DOI:** 10.1186/s40780-024-00356-8

**Published:** 2024-06-26

**Authors:** Hayato Yokota, Ruriko Asahi, Yumiko Akamine, Mizuki Kobayashi, Hiyu Wakabayashi, Sho Sakamoto, Yuji Okuda, Kazuhiro Sato, Katsutoshi Nakayama, Masafumi Kikuchi

**Affiliations:** 1 Department of Pharmacy, Akita University Hospital, 1-1-1 Hondo, Akita, 010-8543 Japan; 2 Department of Respiratory Medicine, Akita University Graduate School of Medicine, Akita, Japan; 3 Department of Cardiovascular Medicine, Akita University Graduate School of Medicine, Akita, Japan

**Keywords:** Anamorelin, Cachexia, Electrocardiogram, Obesity, QTc

## Abstract

**Background:**

Anamorelin, a drug to treat cancer cachexia, binds to ghrelin receptors and improves body weight and appetite. In clinical trials in Japan, patients experienced a 10.7% frequency of stimulant conduction system depression as a severe side effect. Although rare, anamorelin sometimes causes fatal arrhythmias. Because patients with cancer cachexia are often underweight, data on the safety of anamorelin in obese patients are lacking. We report a case of QT interval prolongation after anamorelin administration to an obese patient with non-small cell lung cancer.

**Case presentation:**

A female patient with a body mass index of 30 kg/m2 underwent immunotherapy for lung adenocarcinoma. She presented with severe weight loss, anorexia, and fatigue. She had no history of heart disease. On day 12, after administration of anamorelin 100 mg once daily, the patient developed nausea, diarrhea, and anorexia, which were considered cancer immunotherapy-induced immune-related adverse events, and she was admitted to the hospital. An electrocardiogram (ECG) on admission showed a QTc interval of 502 ms. On admission, her hepatic function was Child–Pugh class B, and anamorelin was discontinued the next day. On day 3 after anamorelin discontinuation, the QTc interval was prolonged by up to 557 ms, then decreased to 490 ms on day 6, and improved to 450 ms on day 16. Re-administration of anamorelin was avoided.

**Conclusions:**

When administering anamorelin to obese patients, we should be aware of the potential for stimulatory conduction system depression, as in underweight patients. Therefore, we should monitor patients by ECG from the early stages of anamorelin administration. Anamorelin is lipophilic, and its volume of distribution is increased in obese patients. Consequently, obese patients may continue to have QT interval prolongation after discontinuation of anamorelin, requiring long-term side-effect monitoring.

## Introduction

Anamorelin is a selective oral ghrelin receptor agonist that improves anorexia and increases lean body mass [1, 2]. Anamorelin was approved in Japan in 2021 for the indication of cancer cachexia in non-small cell lung cancer, gastric cancer, pancreatic cancer, and colorectal cancer, which was its first approval in the world. Anamorelin also has sodium channel-blocking activity. Moreover, anamorelin depresses the stimulant conduction system and may cause marked prolongation of the PR interval, QRS complex, or QT interval. Therefore, anamorelin is contraindicated in patients with congestive heart failure, myocardial infarction or angina pectoris, and severe conduction defects [3]. Many cases of QT interval prolongation are asymptomatic; thus, a diagnosis is rare. However, studies have shown an increased risk of torsade de pointes (TdP) with a QTc > 500 ms [4]. TdP, a type of polymorphic ventricular tachycardia, causes palpitations, dizziness, syncope, and convulsive symptoms. In addition, TdP leads to sudden ventricular fibrillation, which may result in cardiac arrest and sudden death. Several case reports and one letter on cardiac-related adverse events caused by anamorelin have been published. Sakagami and colleagues reported a case of a patient with pancreatic cancer who developed ventricular tachycardia 15 days after starting anamorelin [5]. In addition, a letter was published in which a patient with pancreatic cancer had a prolonged QTc interval of 617 ms on day 20 after anamorelin [6]. Further, Kojima and coworkers reported a case of fatal arrhythmia after the first oral administration of anamorelin in a patient with rectal cancer [7]. In the phase II and phase III clinical trials of anamorelin, adverse reactions related to stimulation conduction system depression were reported in 10.7% of patients [3], and the incidence of adverse events related to stimulation conduction system depression up to day 14 of anamorelin administration was higher than that in other periods. Therefore, focused monitoring by electrocardiogram (ECG) during the early administration stages is essential.

Cancer cachexia is defined as involuntary weight loss > 5% within the past six months or a body mass index (BMI) < 20 kg/m^2^, with weight loss > 2% [8]. In a study examining the prevalence of cachexia in Asian individuals, the average BMI was approximately 22 kg/m^2^ [9]. In the phase III clinical trials (ROMANA 1 and ROMANA 2), the mean BMI was 23.2 ± 3.6 kg/m^2^ and 22.5 ± 3.7 kg/m^2^, respectively [1]. In addition, a multicenter, open-label, single-arm study of anamorelin (ONO-7643) excluded patients with a BMI > 30 kg/m^2^ [10]. Thus, there is a lack of data on the therapeutic efficacy and safety of anamorelin after administration to obese patients. Here, we report a case of QT interval prolongation after anamorelin administration to an obese patient with non-small cell lung cancer.

## Case presentation

The patient was an obese woman in her 50 s with a BMI of 30 kg/m^2^ and a weight of 79 kg who had been diagnosed with lung adenocarcinoma. Her Eastern Cooperative Oncology Group performance status score was 0 before starting treatment. She completed four courses of chemoimmunotherapy, including carboplatin + nab-paclitaxel + atezolizumab, followed by maintenance treatment with atezolizumab monotherapy. The patient developed anorexia and experienced a weight loss of more than 5% in the last six months. Moreover, she met the following criteria for anamorelin initiation: (1) fatigue from cancer cachexia and (2) hemoglobin level < 12 g/dL. She had no history of heart disease. On day 1, anamorelin 100 mg was administered once daily on an empty stomach. At the initiation of anamorelin administration, the patient’s aspartate aminotransferase (AST) level was 22 U/L, alanine aminotransferase (ALT) level was 12 U/L, total bilirubin (T-Bil) was 0.7 mg/dL, serum creatinine (SCr) was 0.76 mg/dL, and serum albumin was 3.9 g/dL.

**Fig. 1:**
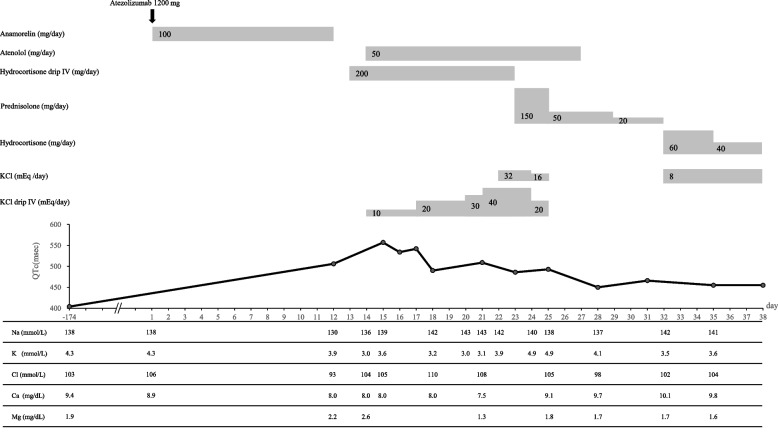
The clinical course of the patient. Doses of atezolizumab, anamorelin, atenolol, hydrocortisone, prednisolone, and KCl are shown. QTc and laboratory parameter trends before and after administration of anamorelin. IV: intravenous infusion; KCl: potassium chloride

**Fig. 2:**
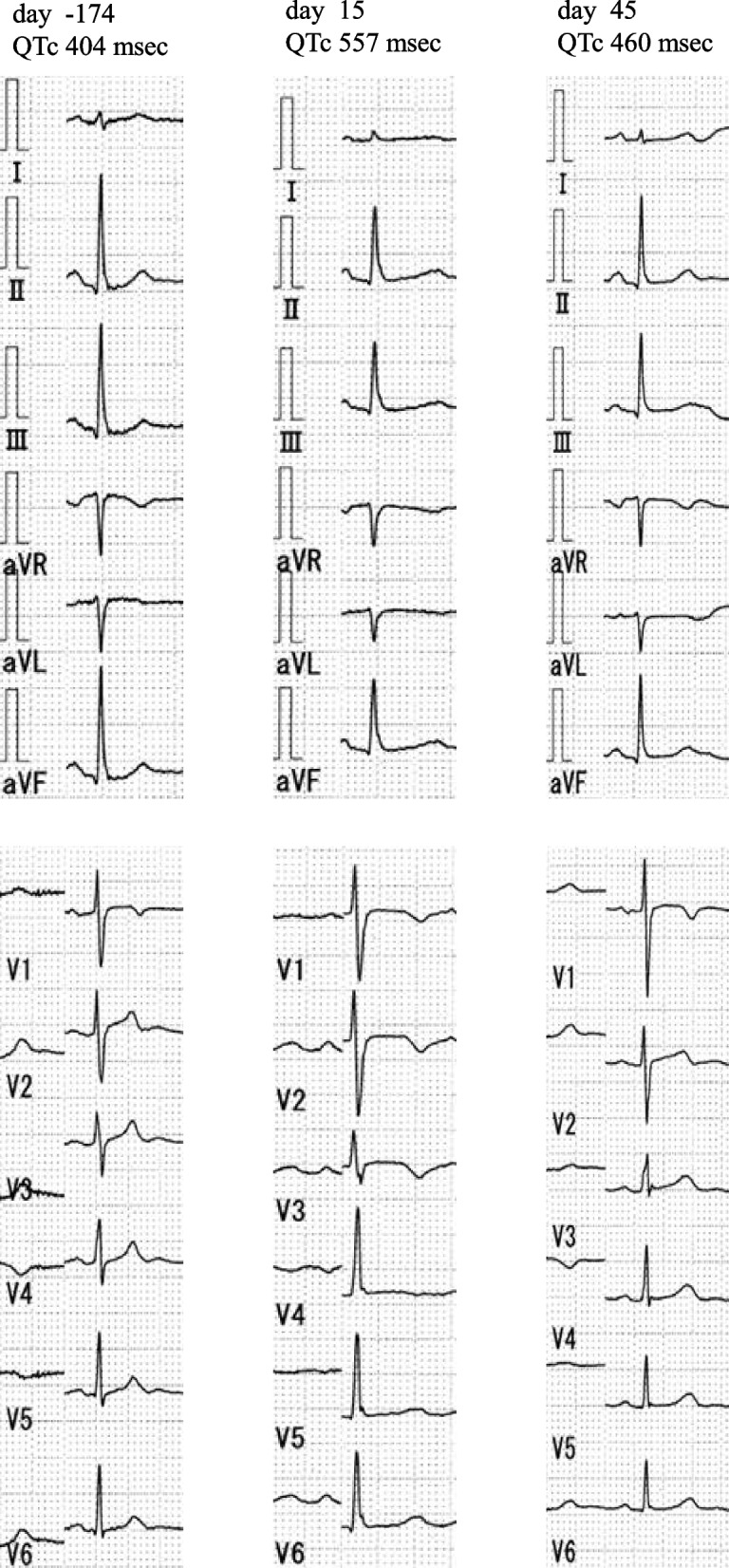
The QTc before the initiation of anamorelin (day -174), during QT prolongation (day 15), and after discontinuation (day 45), along with 12-lead electrocardiograms

On the 12th day after anamorelin administration, the patient developed nausea, diarrhea, and anorexia and was admitted to the hospital. An ECG revealed grade 3 QT prolongation according to the Common Terminology Criteria for Adverse Events version 5.0., with a QTc of 502 ms (Fig. 1). On admission, the patient’s hepatic function was Child–Pugh class B. Blood tests showed AST 46 U/L, ALT 23 U/L, T-Bil 0.8 mg/dL, SCr 0.86 mg/dL, and serum albumin 3.1 g/dL. Drugs used on admission were anamorelin (100 mg/day), rosuvastatin (2.5 mg/day), vonoprazan (10 mg/day), mirogabalin (20 mg/day), and DENOTAS® chewable combination tablets two tablets a day. All oral drugs, including anamorelin, were discontinued the next day, and the patient was fasted. No bradycardia, palpitations, or loss of consciousness were observed, so we followed up with the patient without administering treatment. Meanwhile, the patient was diagnosed with secondary adrenal insufficiency due to isolated adrenocorticotrophic hormone (ACTH) deficiency. Laboratory test results showed low levels of cortisol, ACTH, and thyroid-stimulating hormone (TSH)—1.56 μg/dL, 3.8 pg/mL, and 0.005 μIU/mL, respectively—and high levels of free triiodothyronine (FT_3_) and free thyroxine (FT_4_)—30.3 pg/mL and > 7.77 ng/dL, respectively. The day after discontinuing anamorelin, an intravenous hydrocortisone infusion was initiated at a dose of 200 mg/day. Furthermore, the patient was diagnosed with thyrotoxicosis due to destructive thyroiditis based on thyroid ultrasound findings. Two days after discontinuation of anamorelin, atenolol 50 mg/day was initiated for tachycardia caused by thyrotoxicosis. The patient continued to have diarrhea, which was considered an immune-related adverse event caused by atezolizumab. The patient experienced weight loss and fluctuated in the 67- 73 kg range. The patient was switched from hydrocortisone to intravenous prednisolone 150 mg/day, and a lower gastrointestinal endoscopy was performed. Endoscopic examination revealed no macroscopic abnormalities, and there were no evident abnormalities on histopathological examination. Then, she was allowed to resume eating. Therefore, after prednisolone was tapered, the treatment was switched to oral hydrocortisone 60 mg/day and subsequently tapered. During steroid administration, serum potassium levels decreased, requiring the administration of oral and intravenous potassium preparations. Sixteen days after admission, thyroid hormone levels, T3 and T4, improved to 2.3 pg/mL and 2.22 ng/dL, respectively, and diarrhea showed significant improvement. The TSH level remained low (0.005 μIU/mL); it improved to baseline (4.34 μIU/mL) by discharge. The ECG on day 3 after discontinuation of anamorelin showed sinus rhythm, a QTc interval of 557 ms, and a QRS duration of 106 ms (in Fig. 1, day 15). In addition, the level of cardiac troponin I was 15.6 pg/mL. On day six after discontinuation of anamorelin (in Fig. 1, day 18), the QTc interval had decreased to 490 ms, and on day 16, it had improved to 450 ms (in Fig. 1, day 28). Re-administration of anamorelin was avoided, and the patient was discharged on day 45 after starting anamorelin. Figure 2 shows the 12-lead ECGs before administration, after administration, and after discontinuation of anamorelin.

## Discussion

We report our experience with anamorelin-induced QT interval prolongation in an obese woman. QT prolongation induced by anamorelin occurred on the 12th day after initiation of anamorelin, consistent with existing reports. Patients with cancer cachexia are often underweight, but even in obese patients, we should be aware of the potential side effect of stimulant conduction system depression with anamorelin. Anamorelin is a highly lipophilic drug, with a distribution coefficient (1-octanol/water) of 2.98 [3]. Therefore, in obese patients, there is a possibility of a more extended prolongation of the QT interval over an extended duration due to the increased distribution of anamorelin in the body, as it remains in the body for a long time after discontinuation.

In this patient, we considered that anamorelin inhibits sodium channels, resulting in the prolongation of the QRS complex and QT interval. There has been a case report of wide QRS complex tachycardia induced by anamorelin [11]. In our case, on the third day after anamorelin discontinuation, the QT interval was maximal at 557 ms, and a mild prolongation of the QRS duration of 106 ms was observed.

Many cases of drug-induced QT prolongation are caused by the inhibition of potassium channels [12]. One type of potassium channel is the IKr channel, the pore-forming subunit of which is encoded by the human ether-a-go-go-related gene (hERG). Drug-induced QT prolongation is caused by an inhibitory effect on the hERG channel. In this case, the patient developed immune-related adverse events due to atezolizumab, and initially, we also considered QT interval prolongation as one of its effects. However, it has been reported that monoclonal antibodies, including atezolizumab, have a very low potential to interact with extracellular or intracellular domains on the hERG channel [13]. In addition, similar to other immune checkpoint inhibitors, atezolizumab has not been shown to have a significant effect on the QT profile [14]. In the phase III trial (IMpower130) combining atezolizumab with carboplatin plus nab-paclitaxel chemotherapy as first-line treatment for non-small cell lung cancer, QT interval prolongation was not observed as an adverse event across all grades [15]. Therefore, we considered the contribution of atezolizumab to QT interval prolongation to be minimal. Further, since the patient’s troponin I levels were below the reference value upon admission, we considered myocarditis due to atezolizumab unlikely. In contrast, the inhibitory effect of anamorelin on the hERG channel is broad, with an IC_50_ ranging from 4.3 to 34 μmol/L [16]. The inhibitory effect of anamorelin on hERG channel currents in human cardiomyocytes was reported to have an IC_50_ of 4.3 μmol/L. In contrast, under conditions using HEK293 cells transfected with the hERG gene, it was reported to be 34 μmol/L. The risk of causing TdP is reported to be low when the IC_50_ of drugs exhibiting hERG inhibition exceeds 30 times the maximum plasma concentration of the drug (unbound free drug concentration) [17]. In the case of the anamorelin, with a maximum drug concentration of 629 ng/mL and a protein binding rate of 97.3%–98.3% [3], there is a difference of over 100 times compared with the IC_50_ of 4.3 μmol/L (2350 ng/mL). Therefore, the risk of developing TdP is considered low. Anamorelin, a substrate of the metabolic enzyme cytochrome P450 3A4, may increase blood concentrations in cases of hepatic dysfunction. Okidono and colleagues reported two cases of wide QRS complex tachycardia in patients with Child–Pugh class B hepatic function [11]. The patient had normal liver function at anamorelin initiation and no history of liver disease, such as cirrhosis or cholangitis. However, upon admission (day 12), the patient’s hepatic function was classified as Child–Pugh class B. Therefore, we suggest that the metabolism of anamorelin was impaired, leading to a temporary increase in blood concentration, which led to the prolongation of the QT interval. Anamorelin is a highly lipophilic drug with a distribution coefficient of 2.98. In the halothane-anesthetized guinea pig model, the lipophilicity (logP) of drugs that may prolong the QT interval (e.g., haloperidol, bepridil) is known to correlate well with the heart-to-plasma concentration ratio [18]. These highly lipophilic drugs have been reported to prolong the QT interval in humans [19, 20]. Therefore, given that anamorelin also has high lipophilicity, it is possible that it may cause QT prolongation. The ratio of myocardium to plasma concentrations for antipsychotic drugs known to cause QT prolongation, arrhythmias, and sudden death (such as haloperidol and risperidone) is higher than 4 [21]. The ratio of radioactive concentrations in the heart to plasma 72 h (3 days) after a single oral dose of ^14^C-anamorelin is 4.5 [22]. This suggests that anamorelin has a notable impact on the heart, indicating a substantial tissue distribution to the heart by the third day post-administration. Obesity is recognized as a predictor for sudden cardiac death, further contributing to increased QTc and QT or QTc dispersion [23]. Therefore, obesity poses a potential risk of QTc prolongation. Risk factors for drug-induced TdP involve hypokalemia and hypomagnesemia [24], although they were not observed in the blood tests conducted upon admission. The day after discontinuation of anamorelin, hydrocortisone infusion via intravenous drip was initiated for secondary adrenal insufficiency, leading to a decrease in potassium levels and a tendency toward QT interval prolongation. This is considered to be due to a decrease in extracellular potassium concentration, leading to prolonged ventricular repolarization time. Moreover, diarrhea, a thyrotoxic symptom caused by atezolizumab, may have contributed to the decreased potassium levels. Various risk factors for QTc prolongation include BMI ≥ 30 kg/m^2^, hypokalemia (K ≤ 3.5 mmol/L), female gender, age ≥ 65 years, and smoking [25]. Therefore, narrowing down the cause of the QTc prolongation observed in this patient was difficult.

In this case, it took 16 days for the patient's QT interval to return to within normal limits. Anamorelin, being highly lipophilic, may have accumulated in the body over an extended period of time. In patients with higher body weight, the excretion rate of anamorelin tends to increase. However, in obese patients, anamorelin may accumulate in adipose tissue and distribute within the adipose tissue over an extended period of time. It has been reported that obesity contributes to an increase in the volume of distribution (Vd) of drugs in obese patients [26]. Therefore, because the patient was obese, it is possible that the high partition coefficient of anamorelin resulted in a larger distribution volume, leading to prolonged accumulation in the body. Consequently, this may have influenced the time to improve the QT interval.

In the present case, initiating anamorelin during outpatient treatment made it difficult to track detailed changes in lean body mass indicative of treatment effects. In additional, due to the short duration of administration, the treatment effects remained unclear.

In conclusion, we report a case of drug-induced QT interval prolongation due to anamorelin. In obese patients with cancer cachexia, there is a risk of potential QT interval prolongation due to the increased Vd of anamorelin, and these patients may experience stimulatory conduction system depression even after discontinuation of anamorelin. Therefore, it is essential to monitor obese patients, as well as underweight patients, by ECG from the early stages of anamorelin administration.

## Data Availability

Data sharing is not applicable to this article as no datasets were generated or analyzed during the current study.

## Abbreviations

ACTH: Adrenocorticotrophic hormone
ALT: Alanine aminotransferase
AST: Aspartate aminotransferase
BMI: Body mass index
ECG: Electrocardiogram
FT_3_: Free triiodothyronine
FT_4_: Free thyroxine
hERG: Human ether-a-go-go-related gene
SCr: Serum creatinine
T-Bil: Total bilirubin
TdP: Torsade de pointes
TSH: Thyroid-stimulating hormone
Vd: Volume of distribution
